# Cryo-EM structure of the *Ustilago maydis* kinesin-5 motor domain bound to microtubules

**DOI:** 10.1016/j.jsb.2019.07.003

**Published:** 2019-09-01

**Authors:** Ottilie von Loeffelholz, Carolyn Ann Moores

**Affiliations:** Institute of Structural and Molecular Biology, Birkbeck College, London WC1E 7HX, UK

**Keywords:** AMPPNP, adenosine 5′-(β,γ-imido)triphosphate, CNB, cover neck bundle, cryo-EM, cryo-electron microscopy, MT, microtubule, N+UmKin5, *U. maydis* kinesin-5 motor domain with extended N-terminus, UmKin5, *U. maydis* kinesin-5 motor domain, 3D reconstruction, Cryo-electron microscopy, Cytoskeleton, Kinesin-5, Microtubule

## Abstract

In many eukaryotes, kinesin-5 motors are essential for mitosis, and small molecules that inhibit human kinesin-5 disrupt cell division. To investigate whether fungal kinesin-5s could be targets for novel fungicides, we studied kinesin-5 from the pathogenic fungus *Ustilago maydis*. We used cryo-electron microscopy to determine the microtubule-bound structure of its motor domain with and without the N-terminal extension. The ATP-like conformations of the motor in the presence or absence of this N-terminus are very similar, suggesting this region is structurally disordered and does not directly influence the motor ATPase. The *Ustilago maydis* kinesin-5 motor domain adopts a canonical ATP-like conformation, thereby allowing the neck linker to bind along the motor domain towards the microtubule plus end. However, several insertions within this motor domain are structurally distinct. Loop2 forms a non-canonical interaction with α-tubulin, while loop8 may bridge between two adjacent protofilaments. Furthermore, loop5 – which in human kinesin-5 is involved in binding allosteric inhibitors – protrudes above the nucleotide binding site, revealing a distinct binding pocket for potential inhibitors. This work highlights fungal-specific elaborations of the kinesin-5 motor domain and provides the structural basis for future investigations of kinesins as targets for novel fungicides.

## Highlights

• The *Ustilago maydis* kinesin-5 N-terminus is disordered in cryo-EM reconstructions.
• AMPPNP-bound *U. maydis* kinesin-5 motor adopts a canonical ATP-like conformation.
• Fungal-specific inserts form non-canonical contacts with the microtubule.
• *U. maydis* kinesin-5 loop5 forms a distinct binding pocket for potential inhibitors.

## Graphical abstract

**Figure:**
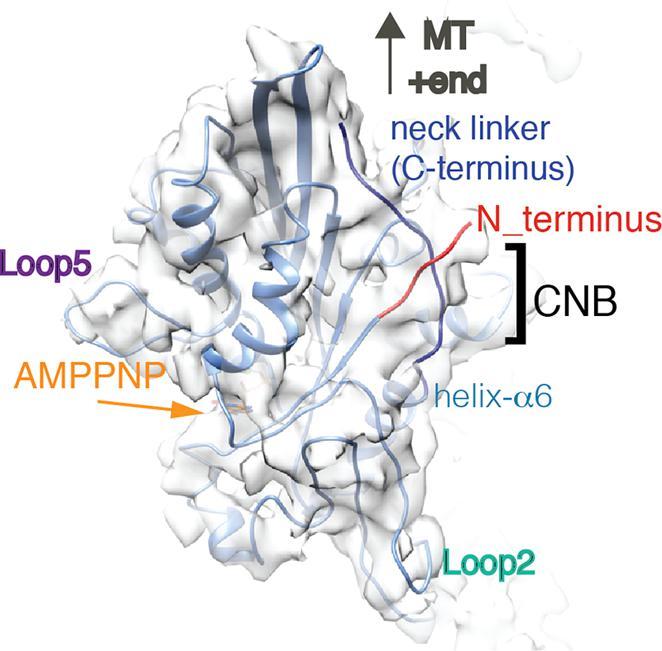

## 1 Introduction

Fungi are significant and increasing mediators of pathogenesis, and cause challenges medically (e.g. *Candida* spp.; *A. fumigatus*), environmentally (e.g. ash dieback-causing *H. fraxinea*) and economically (e.g. rice blast, *M. oryzae*; honeybee colony collapse, *Nosema* spp.) (Bougnoux et al., 2018, Fisher et al., 2012). *Ustilago maydis* is an invasive filamentous fungus that causes corn smut, in which infections stunt plant growth, reduce crop yields and thereby represent a threat to food security (Steinberg, 2007). Emerging resistance to current fungicides means that novel, fungal-specific small molecule targets are urgently needed.

Kinesins are microtubule (MT)-based ATP-driven motors that have many important roles in eukaryotes. This includes the essential activities of several members of the superfamily in cell division (Cross and McAinsh, 2014). Kinesin-5 motors are important for mitosis in many organisms and, for example, functional disruption of kinesin-5s in fungi and vertebrates prevents formation of the bipolar spindle (Goulet and Moores, 2013). Kinesin-5s have been of additional interest because allosteric inhibitors of human kinesin-5 block mitosis, which have thus formed the basis of drug discovery programmes for cancer treatments (Rath and Kozielski, 2012).

Blockage of mitosis through kinesin-5 inhibition could have wider applications in disease control, for example in killing eukaryotic pathogens. As is seen for kinesin-5 in other fungi (Hagan and Yanagida, 1992, Hoyt et al., 1992), *Ustilago maydis* kinesin-5 localizes to mitotic spindles and contributes to spindle elongation at the start of anaphase (Fink et al., 2006). However, mechanistic information is lacking about the extent of conservation of kinesin-5 molecular mechanism across eukaryotes, and therefore whether any structural or mechanistic differences could be exploited to selectively inhibit mitosis in pathogenic organisms.

Kinesins are defined by their conserved motor domains, which contain both ATP- and MT-binding sites, and to which all known inhibitors bind in human kinesin-5. Subtle modifications of sequence and structure within the motor domains can have dramatic impacts on the properties of individual motors and their susceptibility to inhibition (Behnke-Parks et al., 2011, Liu et al., 2011, von Loeffelholz et al., 2019). To begin to understand whether *Ustilago maydis* kinesin-5 is a possible target for novel and specific fungicides, we determined the structures of two different motor domain constructs of this fungal kinesin – with and without its fungal-specific N-terminal extension - bound to MTs using cryo-electron microscopy (cryo-EM). Both proteins were trapped bound to MTs in their ATP-like conformation using the non-hydrolysable analogue AMPPNP. Their structures were determined at an overall resolution of 4.5–5 Å, while the kinesin motor domains have a slightly lower resolution of 5–7 Å (Fig. S1A–D). The structures of this fungal motor allow visualization of conserved aspects of kinesin mechanochemistry and of divergent aspects of its interaction with MTs.

## 2 Materials and methods

### 2.1 Protein expression, purification and preparation

Using a full-length clone gifted by Prof. Gero Steinberg (University of Exeter, UK), two *Ustilago maydis* kinesin-5 motor domain constructs – N+UmKin5 (residues 1–456) and UmKin5 (residues 73–456) – with and without the N-terminal extension respectively, were PCR amplified and cloned into a pNIC28BsaI vector (Structural Genomics Consortium, Oxford), and the recombinant His_6_-tagged monomeric constructs were expressed in BL21*(DE3) *Escherichia coli* cells. Cells were grown in LB medium, supplemented with 2% (w/vol) glucose. Protein expression was induced by addition of 1 mM isopropyl β-D-1-thiogalactopyranoside (IPTG) at 18 °C for 5 h. Cells were resuspended in buffer A (50 mM Tris, 400 mM NaCl, 2 mM MgCl_2_, 5 mM 2-mercaptoethanol, 1 mM ATP) with protease inhibitors (Roche), and lysed using a French press. The N-terminally His_6_-tagged proteins were purified from the clarified cell supernatant using nickel affinity chromatography and the His_6_ tag was removed using TEV protease during overnight dialysis into buffer A. Immediately prior to use, the purified proteins were buffer exchanged into BrB25+ (25 mM PIPES pH 6.8, 30 mM NaCl, 1 mM MgCl_2_, 1 mM EGTA, 1 mM 2-mercaptoethanol) and 5 mM AMPPNP using a Vivaspin® column (Sartorius).

### 2.2 Sample preparation for cryo-EM

MTs were polymerized from bovine brain tubulin (Cytoskeleton, Inc.), at a final concentration of 5 mg/mL, at 37 °C for 1.5 hr in 100 mM MES pH 6.5, 1 mM MgCl_2_, 1 mM EGTA, 1 mM DTT and 5 mM GTP buffer. MTs were stabilized with 1 mM paclitaxel (Calbiochem) in DMSO with incubation for a further 1.5 hr at 37 °C. 100 μM of each motor domain construct was mixed with 14 μM stabilized MTs, and 4 μl of this mixture was applied onto Quantifoil R 2/2 holey carbon grids glow-discharged in air, which were blotted and plunge frozen into liquid ethane using a Vitrobot IV (FEI/Thermo Fisher) operating at room temperature and 100% humidity.

### 2.3 Cryo-EM data collection, structure determination and modelling

Low dose movies were collected manually on a 300 kV Tecnai G2 Polara (FEI) microscope equipped with a Quantum energy filter and K2 Summit direct electron detector (Gatan) in counting mode, with a total dose in each of 30 e^−^/Å^2^ fractioned into 50 frames at a pixel size of 1.39 Å/px. 364 and 284 movies were collected for N+UmKin5 and UmKin5 respectively. Initial frame alignment was performed using IMOD (Kremer et al., 1996). A second local alignment step was performed with Scipion using the optical flow method (de la Rosa-Trevin et al., 2016). The final reconstruction of MTs decorated with N+UmKin5 was calculated from particles containing all frames resulting in a total dose of 30 e^−^/Å^2^, while for the structure of MTs decorated with UmKin5, only frames 2–21 were included resulting in a total dose of 12 e^−^/Å^2^.

For the N+UmKin5-MT dataset, 8210 MT segments were selected in 908 Å^2^ boxes in Boxer (Ludtke et al., 1999) using the helix option and choosing an overlap that left three tubulin dimers (240 Å) unique in each box. Of the 785 MTs that were initially boxed from 364 motion-corrected movies, 397 MTs with 13_3 architecture were selected. The final 3D-reconstruction was generated from 164,177 asymmetric units using a previously described semi-automated single particles approach for pseudo-helical assemblies in SPIDER and FREALIGN (Sindelar and Downing, 2007). The initial reference was a 15 Å low-pass filtered synthetic map generated from an atomic model of a kinesin motor domain- bound MT (Sindelar and Downing, 2007). In the subsequent refinement round, the reference was low-pass filtered to 15 Å, and in the remaining three refinement rounds to 10 Å. The final reconstruction was automatically B-factor sharpened in RELION with a calculated B-factor of −124 (Scheres, 2012), a mask generated in RELION was applied at a threshold such that all parts of the density were included - widened by 3 pixels and a 3 pixels soft edge - and the sharpened map was subsequently filtered to 5.0 Å. The final overall resolution of the masked reconstruction was 4.5 Å (0.143 FSC cutoff, 5.9 Å using 0.5 FSC cutoff, Fig. S1A). For the UmKin5-MT dataset, 9520 MT segments were selected as for N+UmKin5. Of the 672 MTs that were initially boxed from 284 motion-corrected movies, 306 MTs with 13_3 architecture were selected. The final 3D-reconstruction was calculated from 176,553 asymmetric units and calculated as for N+UmKin5. The final reconstruction was automatically B-factor sharpened in RELION with a calculated B-factor of −154 (Scheres, 2012), a mask generated in RELION was applied at a threshold such that all parts of the density were included (as above) and the sharpened map was subsequently filtered to 4.5 Å. The final overall resolution of the masked reconstruction was 4.3 Å (0.143 FSC cutoff, 5.8 Å using 0.5 FSC cutoff, Fig. S1D). Local resolution was calculated using RELION (Scheres, 2012), and structures were visualized using Chimera (Pettersen et al., 2004).

As is typical in motor-MT complexes (Kellogg et al., 2017), there is a resolution gradient from MT to kinesin density (Fig. S1B,E). The average resolution in the tubulin region of the reconstructions is ~4.4 Å (Fig. S1C,F) while resolution of the (N+)UmKin5 is ~5–7 Å. Guided by the local resolution, we focused on the interpretation of secondary structure information for UmKin5, while the previous published coordinates of tubulin (3J6G, (Alushin et al., 2014)) were used to model the MT part of the reconstruction.

A homology model for UmKin5 was generated using a crystal structure of the human kinesin-5 motor domain bound to AMPPNP (3HQD, (Parke et al., 2010)) using Chimera (Pettersen et al., 2004). The model was adjusted to the calculated cryo-EM maps using Coot (Emsley et al., 2010), taking into account density at lower thresholds in flexible regions, e.g. loop 2, and was subjected to real space refinement in Phenix (Adams et al., 2010) using the EM-derived density filtered to 4.5 Å (see Table 1).

**Table 1:** Refinement statistics and model geometry for the UmKin5 and N+UmKin5 models.

	UmKin5	N+UmKin5
RMSD (bonds)	0.01	0.00
RMSD (angles)	1.04	0.86
All atoms clashscore	11.88	9.74
Ramachandran outliers	0.00%	0.08%
Ramachandran allowed	10.83%	7.00%
Ramachandran favoured	89.17%	92.92%
Rotamer outliers	0.10%	0.00%
Refinement resolution (Å)	4.5	5

## 3 Results and discussion

### 3.1 Overview of the structure of MT-bound *U. maydis* kinesin-5 motor domain

The motor domains from a number fungal kinesin-5s contain sequence insertions within the canonical kinesin fold. In addition, these motors often carry extended N-terminal regions, some of which bind MTs independently of the motor domain (Britto et al., 2016, Stock et al., 2003). Like BimC from *A. nidulans* and Cut7 from *S. pombe*, *U. maydis* kinesin-5 has a 72 residue N-terminal extension (Fig. S2). We therefore undertook the structural characterization of two *U. maydis* kinesin-5 motor domain constructs with and without this N-terminal extension: N+UmKin5 and UmKin5. Both constructs bound the MT lattice in the presence of the non-hydrolysable ATP analogue AMPPNP, allowing us to calculate their 3D structures.

**Fig. 1:**
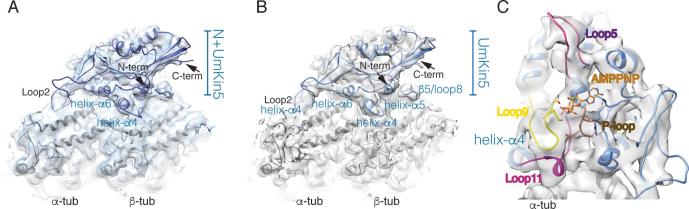
Cryo-EM reconstructions of MT-bound *U. maydis* kinesin-5 motor domain constructs in their ATP-like state. A) The asymmetric unit of MT-bound N+UmKin5 viewed with the MT plus end to the right. The ribbon model corresponds to the motor domain and fits the density well, indicating that there is no additional density corresponding to its N-terminal extension; B) The asymmetric unit of MT-bound UmKin5 viewed with the MT plus end to the right. The ribbon model corresponds to the motor domain and is very similar to that of N+UmKin5, reinforcing the observation that no additional density corresponding to the motor N-terminus is visible in the N+UmKin5 reconstruction; C) View of the UmKin5 nucleotide binding pocket showing helix-α4 at the back of the pocket, the conserved nucleotide-binding loops - P-loop (brown), loop9 (yellow) and loop11 (magenta) – as well as loop5 (pink) which emerges above the nucleotide binding site.

The asymmetric unit of these reconstructions is the motor domain bound to an αβ-tubulin heterodimer (Fig. 1A, B). Although the density observed depends on the overall resolution of the reconstruction and the display threshold used, little interpretable density is visible in the N+UmKin5 reconstruction beyond the N-terminus of the motor domain itself, i.e. our structures do not provide any structural information about the *U. maydis* kinesin-5 N-terminal extension (Fig. 1A). This was also the case in reconstructions of MTs bound by the Cut7 motor domain (Britto et al., 2016), and is presumably because the N-terminus does not adopt a regular structure and cannot be visualized in our calculated structure. Since the MT-bound reconstructions of N+UmKin5 and UmKin5 are essentially indistinguishable, we present and describe below the higher resolution UmKin5-MT reconstruction (Fig. 1B).

As is typical for kinesin motor domains, the UmKin5 density is split into three layers: a central β-sheet with sets of α-helices on either side (Fig. 1B). UmKin5 binding to the αβ-tubulin is centred on helix-α4. Additional contacts with the MT surface are formed by helix-α5, β5/loop8 on β-tubulin, and loop2 on α-tubulin (Fig. 1B). Strong density is visible at the nucleotide binding site consistent with the presence of AMPPNP. The bound nucleotide is surrounded by highly conserved sequences in the kinesin motor domain corresponding to the P-loop (Fig. 1C, in brown), loop 9, which contains the switch I sequence (Fig. 1C, in yellow), and loop 11, which contains the switch II sequence (Fig. 1C, in magenta; Fig. S2). Below the nucleotide binding site, the helical turn in loop11 that leads into helix-α4 is associated with the surface of α-tubulin in this state. Based on prior knowledge about the response of many kinesin motor domains to AMPPNP, the nucleotide binding site loops in UmKin5 have been modelled to adopt a compact conformation (Britto et al., 2016, Parke et al., 2010, von Loeffelholz et al., 2019).

### 3.2 Formation of a cover neck bundle in UmKin5 is consistent with plus end directed motility

**Fig. 2:**
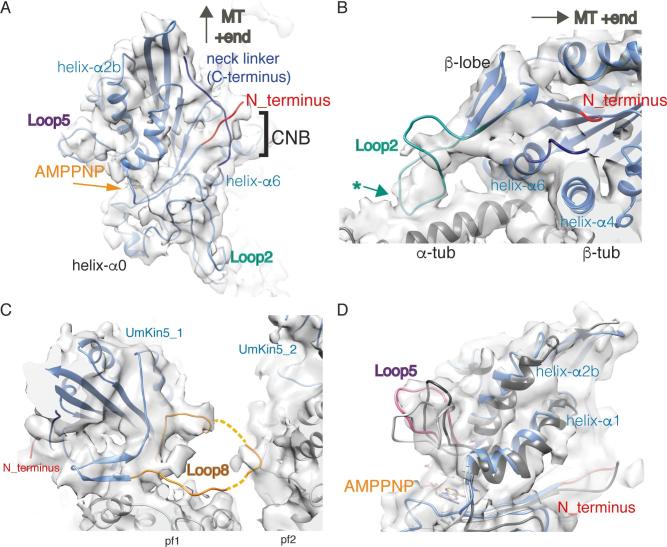
Canonical ATP-like state of MT-bound *U. maydis* kinesin-5 motor domain and distinct conformations of loop insertions. A) UmKin5 cover neck bundle (CNB) formation from the docked neck linker (blue) – directed towards the MT plus end - and the N-terminus (red); B) The UmKin5 loop2 insertion (turquoise) emerges from the minus end of UmKin5 and forms a contact (*) with α-tubulin; C) The UmKin5 loop8 insertion (orange) is long enough to contact β-tubulin in the adjacent protofilament; however, density is incomplete in this region of the reconstruction (dashed lines), suggesting such connectivity is flexible; viewed from the MT plus end, adjacent protofilaments are labelled pf1 and pf2, with each pf bound by a UmKin5 molecule (UmKin5_1, UmKin5_2); D) Close up view of the distinct conformation of UmKin5 loop5 (pink) protruding from the motor domain above the nucleotide binding pocket, compared to that of Cut7 (dark grey, PDB: 5MLV) and human kinesin-5 (light grey, PDB: 3HQD).

The N- and C-termini emerge from UmKin5 adjacent to each other on the side of the motor domain opposite the nucleotide binding site (Fig. 2A). Based on the well-defined position of helix-α6 relative to helix-α4 (Fig. 1A, B), and in the context of extensive prior knowledge about the consequences of this configuration for the conformation of the motor N- and C-termini (Britto et al., 2016, Parke et al., 2010, von Loeffelholz et al., 2019), density visible along the edge of the motor domain central β-sheet is consistent with docking of the motor’s C-terminal neck linker, directed towards the MT plus-end (Fig. 2A). This structural response to AMPPNP is consistent with the idea that UmKin5 can move towards the MT plus end. Furthermore, although most of the N-terminus is absent in the UmKin5 construct and not visible in the N+UmKin5 reconstruction, density corresponding to the N-terminus proximal to the motor domain is visible in both structures (Fig. 1A, B). Our pseudo-atomic model suggests that they lie above the neck linker adjacent to the C-terminus of helix-α4 and form the so-called cover neck bundle (CNB, Fig. 2A), which is likely to contribute to force generation in UmKin5 (Hwang et al., 2008, Khalil et al., 2008).

### 3.3 Visualization of UmKin5 specific loops

Loop2 is longer in UmKin5 than human kinesin-5 and Cut7 (Fig. S2), and density corresponding to this loop emerges from a small β-sheet lobe at the motor domain minus end, forming an apparently somewhat flexible contact with the surface of α-tubulin (Figs. 1A, B, 2B). This connectivity is not seen in other kinesin-5s (Goulet et al., 2012, von Loeffelholz et al., 2019), nor in mammalian transport kinesins, like kinesin-1 and kinesin-3 (Atherton et al., 2014, Shang et al., 2014). The importance of the loop2-MT interaction is best described in the context of the depolymerization activity of kinesin-13s (Ogawa et al., 2004, Shipley et al., 2004), while contacts between α-tubulin and loop2 of mammalian kinesin-8s (Locke et al., 2017, Wang et al., 2016) are important either for accumulation at kinetochore MT ends (Kim et al., 2014) or for depolymerisation activity (Wang et al., 2016). Loop2 of kinesin-6 MKLP2 also contacts α-tubulin (Atherton et al., 2017) but its contribution to this motor’s function is not known. Overall, connectivity between loop2 and the MT is linked in many, but not all, kinesins to regulation of MT dynamics. No evidence of MT destabilization was seen in the EM samples of either N+UmKin5 and UmKin5 bound to MTs and this loop could, instead, play a role in stabilizing the motor-MT interaction. Further, future work will be necessary to examine the role of loop2 in *U. maydis* kinesin-5 function.

Loop8 – which is also longer in UmKin5 than human kinesin-5 and Cut7 (Fig. S2) – protrudes from the opposite side of the motor domain to loop2. Even at lower thresholds, density corresponding to loop8 is incomplete, consistent with flexibility in this region (dashed lines, Fig. 2C). However, loop8 is positioned, and is long enough, to form a flexible additional tether from the motor domain to β-tubulin in an adjacent protofilament. Such a linkage could facilitate motor processivity or cooperativity.

Loop5 emerges from above the nucleotide binding site and, in the case of UmKin5, protrudes away from the motor domain, similar to the conformation in Cut7 loop5 (Fig. 2D) (von Loeffelholz et al., 2019). This configuration contrasts with that seen in human kinesin-5 (Fig. 2D), where loop5 curves from helix-α2 towards helix-α3 to form the binding site for many human kinesin-5-specific allosteric inhibitors (Rath and Kozielski, 2012). The specific conformation of loop5 is critical in allowing these inhibitors to bind (Behnke-Parks et al., 2011, Liu et al., 2011, von Loeffelholz et al., 2019), suggesting that UmKin5 could be selectively inhibited by a different range of small molecules.

Overall, our structures show that the ATP-like state of UmKin5 is similar to that observed for other plus-end directed kinesins, but also highlight non-canonical features of this motor.

Future work characterising UmKin5 activity will be important to place this structural information in functional context and to establish for example whether UmKin5, like other fungal kinesin-5s, is capable of switching directions on MTs (Britto et al., 2016, Edamatsu, 2014, Gerson-Gurwitz et al., 2011, Roostalu et al., 2011). It may also be of interest to understand the influence of fungal-derived MTs on motor activity, since they may exhibit some divergent properties compared with the mammalian tubulin MTs used in the current study (von Loeffelholz et al., 2019, von Loeffelholz et al., 2017). Our study of UmKin5 provides structural support for our hypothesis that divergent inserts in fungal kinesin-5 motor domains may facilitate specification for novel fungicides against pathogenic fungi, and future studies will be directed towards investigating this further.

### 3.4 Data deposition

The cryo-EM reconstructions that support the findings of this study has been deposited in the Electron Microscopy Data Bank, accession numbers 3529 (UmKin5) and 3530 (N+ N+UmKin5). The docked coordinates reported in this paper have been deposited in the Protein Data Bank, www.pdb.org, accession numbers 5MM4 (UmKin5) and 5MM7 (N+UmKin5).

## Declaration of Competing Interest

None.
