# Supplementary material for: Cryo-EM structure of the Ustilago maydis kinesin-5 motor domain bound to microtubules
Source: J Struct Biol. 2019 Sep 1;207(3):312–6. doi: 10.1016/j.jsb.2019.07.003 (PMC6722389; doi:10.1016/j.jsb.2019.07.003)
Supplement: Supplementary data 1 [file mmc1.pdf]

# Supplementary Data

# Cryo-EM structure of the *Ustilago maydis* kinesin-5 motor domain bound to microtubules

Ottilie von Loeffelholz[1,2] and Carolyn Ann Moores[1*]

[1] Institute of Structural and Molecular Biology, Birkbeck College, London WC1E 7HX, U.K.
[2] Current address: Centre for Integrative Biology, Department of Integrated Structural Biology, Institute of Genetics and of Molecular and Cellular Biology, 1 rue Laurent Fries, 67404 Illkirch, France; Centre National de la Recherche Scientifique UMR 7104, Illkirch, Université de Strasbourg, Strasbourg, France; INSERM U964, Illkirch, Université de Strasbourg, Strasbourg, France.

* Corresponding Author
Carolyn A. Moores; ORCID ID: 0000-0001-5686-6290
E-mail: c.moores@mail.cryst.bbk.ac.uk

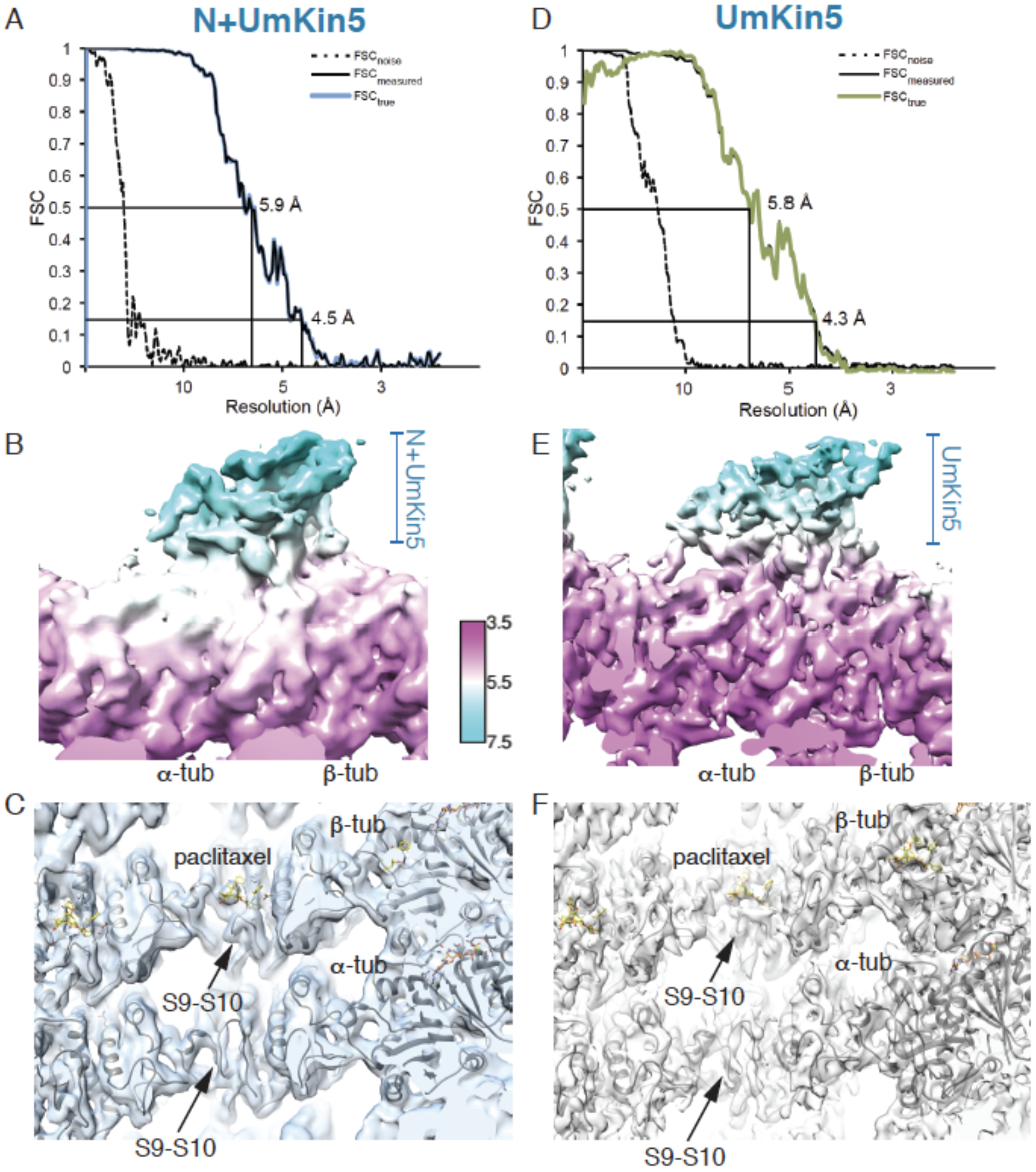


**Figure S1. Evaluation of the (N+)UmKin5-MT cryo-EM reconstructions.**
A) FSC curve for the overall N+UmKin5-MT reconstruction with resolution estimated to be 4.5 Å by the 0.143 criterion and $FSC_{true}$ curves were calculated according to Chen et al (2013); B) Depiction of local resolution estimate in the reconstruction using RELION, which shows the resolution gradient between the MT and the kinesin motor domain; C) View of the tubulin density from the MT lumen, highlighting that the quality of the density in this region of the reconstruction allows differentiation between α- and β-tubulin by the lengths of loop S9-S10 in each monomer, and by density corresponding to paclitaxel bound to β-tubulin, which stabilizes the MTs; D) FSC curve for the overall UmKin5-MT reconstruction with resolution estimated to be 4.3 Å by the 0.143 criterion; E) Depiction of local resolution estimate in the reconstruction using RELION; F) View of the tubulin density from the MT lumen, highlighting differences between α- and β- loops S9-S10, and density corresponding to paclitaxel bound to β-tubulin.

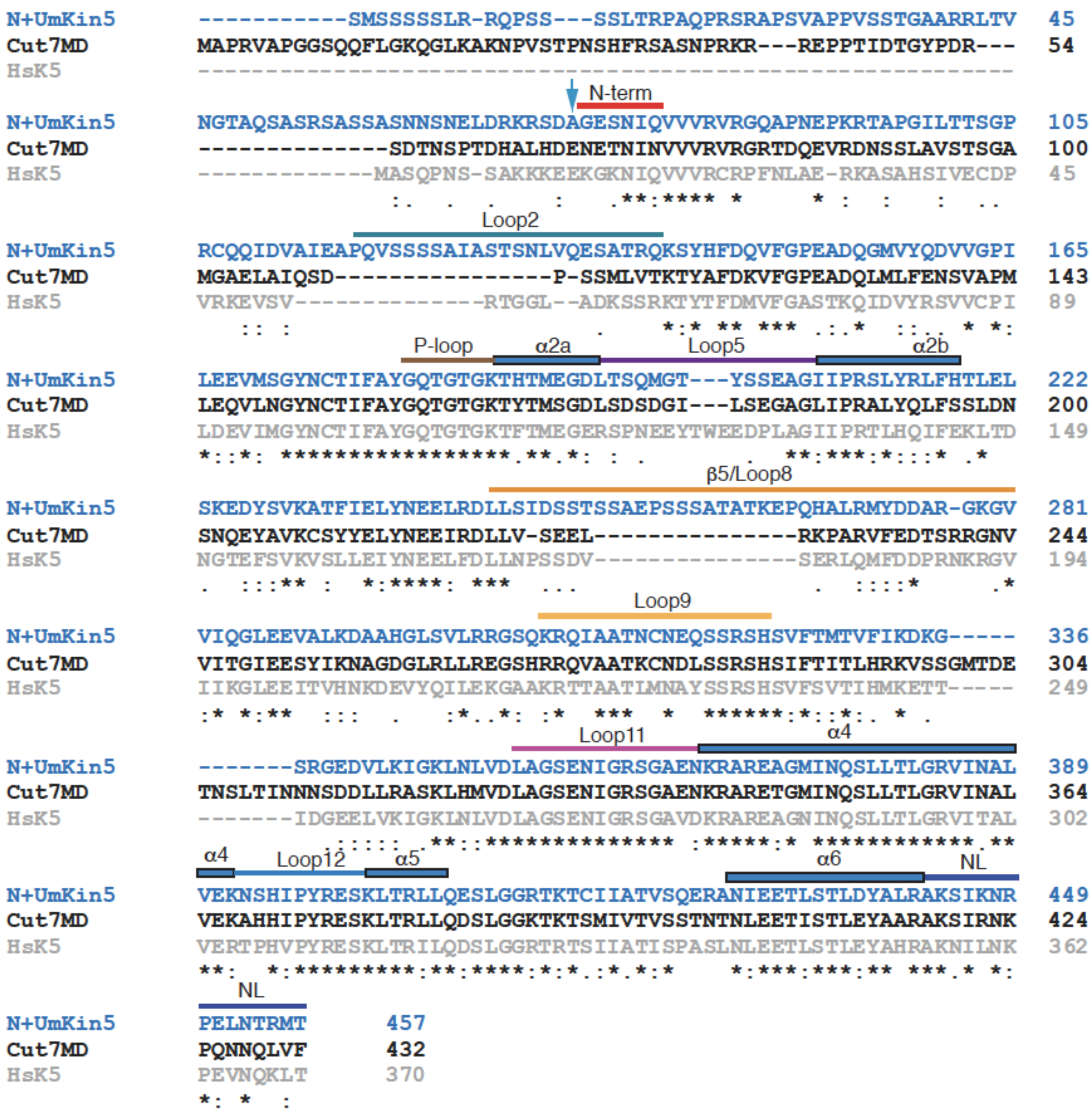


**Figure S2. Sequence alignment of UmKin5, Cut7 and human Kif11 kinesin-5 motor domains.** Motor domain sequences (UmKin5: Uniprot: A0A0D1DQH0_USTMA; Cut7: Uniprot: P24339; human Kif11: Uniprot: P52732) were aligned using Clustal Omega (Sievers et al., 2011) with sequence conservation depicted below the alignment. Secondary structure elements referenced in the text are annotated (NL, neck linker), and the N-terminus of the UmKin5 construct used in this study is indicated with an arrow.

## References


Chen, S., McMullan, G., Faruqi, A.R., Murshudov, G.N., Short, J.M., Scheres, S.H., and Henderson, R. (2013). High-resolution noise substitution to measure overfitting and validate resolution in 3D structure determination by single particle electron cryomicroscopy. Ultramicroscopy *135*, 24-35.

Sievers, F., Wilm, A., Dineen, D., Gibson, T.J., Karplus, K., Li, W., Lopez, R., McWilliam, H., Remmert, M., Soding, J., *et al.* (2011). Fast, scalable generation of high-quality protein multiple sequence alignments using Clustal Omega. Mol Syst Biol *7*, 539.
